# A novel mutation of laminin β2 (*LAMB2)* in two siblings with renal failure

**DOI:** 10.1007/s00431-017-2871-6

**Published:** 2017-02-10

**Authors:** Farah A. Falix, Carlien A.M. Bennebroek, Bert van der Zwaag, Ruth Lapid-Gortzak, Sandrine Florquin, Michiel J.S. Oosterveld

**Affiliations:** 1 Pediatric, University Medical Center Utrecht, Utrecht, The Netherlands; 2 Ophthalmology, University Medical Center Utrecht, Utrecht, The Netherlands; 3 Genetics, University Medical Center Utrecht, Utrecht, The Netherlands; 4 Pathology of the Emma Children’s Hospital/Academic Medical Center, University Medical Center Utrecht, Utrecht, The Netherlands

**Keywords:** *LAMB2*, Pierson syndrome, Nephrotic syndrome, Ocular abnormalities

## Abstract

This report describes a novel mutation of *LAMB2*, the gene associated with Pierson syndrome (microcoria-congenital nephrosis syndrome), in two female siblings. The c.970T>C p.(Cys324Arg) mutation in the *LAMB2* gene affects one of the eight highly conserved cysteine residues within the first EGF-like module of the laminin β2 protein. These residues form disulfide bonds in order to achieve a correct 3D structure of the protein. The reported phenotype is considered a relatively mild variant of Pierson syndrome and is associated with later-onset (18 months) therapy-resistant nephrotic syndrome leading to renal failure, and ocular abnormalities consisting of high myopia, microcoria, diverse retinal abnormalities, hence a low level of visual acuity. Importantly, the reported *LAMB2* mutation was associated with normal neurological development in both siblings.

*Conclusion* ***:*** this report presents the variability of the renal, ocular and neurological phenotypes associated with *LAMB2* mutations and underscores the importance of ophthalmologic examination in all children with unexplained renal insufficiency or nephrotic syndrome.

**Table:** 

**What is known**
• *LAMB2* mutations are associated with Pierson syndrome
• Pierson syndrome is associated with congenital nephrotic syndrome, microcoria and neurological deficits
**What is new**
• A novel mutation in the *LAMB2* gene in two female siblings
• Genotype and clinical phenotype description of a novel *LAMB2* mutation

## Abbreviations

*LAMB2*Laminin β2

OCTOptic coherence tomography

## Introduction

Mutations in the laminin β2 (*LAMB2)* gene are associated with Pierson syndrome [1]. In 2004, this rare autosomal recessive disorder was first described in patients suffering from congenital nephrotic syndrome (i.e., presenting before the age of 3 months) with rapid progression to end-stage renal disease (ESRD), combined with distinct ocular malformations and neurodevelopmental deficits. Ocular manifestations included microcoria, abnormal lens shape with cataracts, and retinal abnormalities [1, 2]. In the last decade, milder phenotypes associated with various *LAMB2* mutations have been reported [2–6]. The *LAMB2* gene is located on chromosome 3 and encodes the laminin β2 protein belonging to the laminin family of extracellular matrix glycoproteins, which are the major non-collagenous constituents of basement membranes. They have been implicated in a wide variety of biological processes [7]. Laminins represent a group of cross-shaped heterotrimeric proteins consisting of α, β, and γ subunits joined together to form a 3D-coiled structure [4, 8]. The human *LAMB2* gene is composed of 32 densely packed exons and encodes a protein of 1798 amino acids [4, 7, 8]. The laminin β2 protein is the major laminin component of the glomerular basement membrane and is also involved in ocular and neuromuscular synapse development [2, 7]. It has been postulated that the severe phenotype, as originally described by Pierson, is associated with truncating *LAMB2* mutations leading to a complete loss of laminin β2 expression and that milder phenotypes reflect mutations with residual laminin β2 function [3, 4]. In this report, we describe a novel mutation in the *LAMB2* gene and its associated phenotype.

## Case reports

### Medical history

Two female siblings, 5 and 6 years old, were admitted to our pediatric nephrology department for renal failure of unknown cause. They were the only two children of consanguineous parents from Syria. On admission, both girls were severely nutritionally deprived, growth retarded—and they suffered from dental caries.

#### Case 1

The eldest girl had presented with steroid-resistant nephrotic syndrome in Syria at the age of 18 months, followed by deterioration of renal function. Her parents provided a report of a kidney biopsy in 2010, which described mild mesengial glomerulopathy without sclerosis. She had been diagnosed with severe myopia and had completely lost vision in her right eye following a traumatic injury at the age of 4 years. She wore correcting glasses. Following rehydration after admission, her estimated glomerular filtration rate (Schwartz formula) was 15 ml/min/1.73 m [9], corresponding to stage 5 chronic kidney disease.

##### Ocular findings

Ophthalmologic examination revealed blindness of the right eye and low vision of the left eye (best spectacle-corrected distance vision Lea card: Logmar:1.0, Snellen 6/60) without nystagmus. Examination of the right eye showed bandkeratopathy, presumed to be post-traumatic, and mature cataract. Ultrasonography showed a total funnel- shaped retinal detachment. The left eye showed high myopia (spherical equivalent: S-17), microcornea (9 mm diameter), microcoria not responsive to mydriatics, and a clear central lens. Fundus examination showed diffuse tessellation of the retina of the left eye. Due to persistent poor cooperation, information on intraocular pressure, axial length, and appearance of the optic disk could not be obtained. The patient was assisted with low-vision devices and special training for low vision.

In the months after admission, she showed appropriate weight gain following initiation of gastrostomy feeding. She showed normal neurological development and underwent surgery for the creation of an arteriovenous fistula for future hemodialysis.

#### Case 2

The youngest girl had also presented with steroid-resistant nephrotic syndrome at the age of 18 months in Syria. Renal failure in her case was more progressive, with ESRD ensuing at the age of 5 years, for which hemodialysis treatment was initiated in Syria. She had not undergone a kidney biopsy. Like her sister, she wore correcting glasses for high myopia. After admission, clinical evaluation revealed electrolyte imbalances, severe hypertension with left ventricular hypertrophy, and extensive signs of renal osteodystrophy. Despite daily hemodialysis treatment, she showed therapy-resistant hypertension, for which she ultimately required bilateral nephrectomy. Hereafter, her clinical condition gradually improved.

**Fig. 1:**
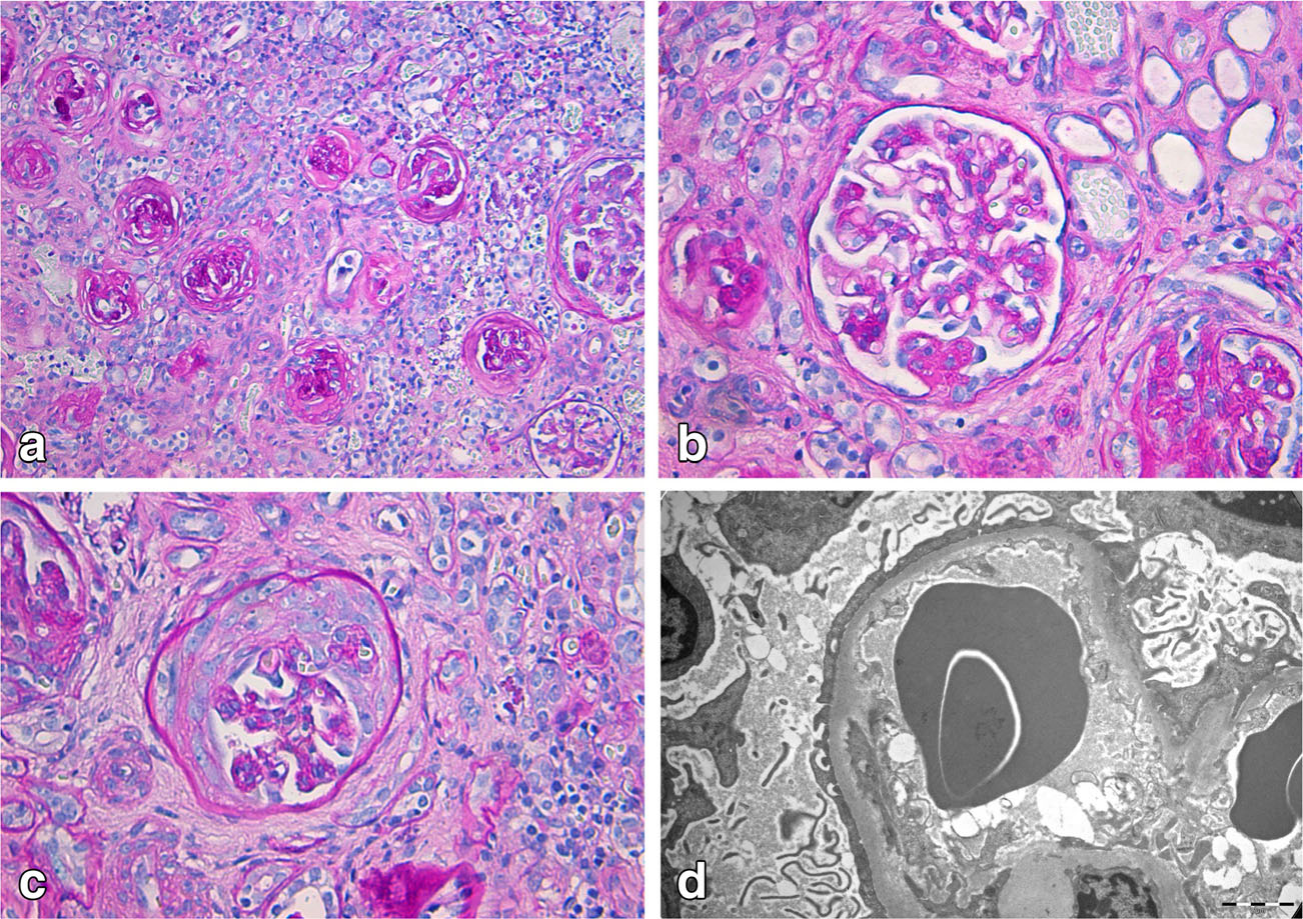
Histopathological findings after nephrectomy in case 2. **a** Representative overview of the renal tissue showing extensive chronic damage with globally sclerotic glomeruli, interstitial fibrosis, inflammation, and tubular atrophy (PAS-diastase staining, ×10). **b** One glomerulus with segmental sclerosis (PAS-diastase staining, ×20). **c** One glomerulus with florid proliferation of parietal epithelial cells (PAS-diastase staining, ×20). **d** Electron microscopy showing irregular glomerular basement membrane and effacement of the podocytes

Histopathological analysis of the nephrectomy showed end-stage kidney disease with signs of a primary podocytopathy consisting of global glomerulosclerosis or severe collapse of the glomerular capillary tuft of more than 90% of the glomeruli (Fig. 1a). A few glomeruli had still open capillaries with segmental sclerosis and adhesion to the Bowman’s capsule (Fig. 1b). Some presented exuberant proliferation of parietal epithelial cells leading to the formation of pseudo-crescents (Fig. 1c). There was very severe atrophy of the tubules with interstitial fibrosis and monocytic inflammatory infiltrates. No specific immune deposits were present on immunofluorescence (data not shown). Electron microscopy showed alteration of the glomerular basement membrane, which appeared irregular with alternating thick and thin zones, and pronounced effacement of the podocytes (Fig. 1d).

##### Ocular findings

Ophthalmologic examination of the youngest sister showed a low level of distance and near visual acuity of both eyes (distance Lea card: Logmar: 1.2, Snellen: 20/125, near Logmar: 0.6, Snellen: 20/80), high myopia (spherical equivalent: S-15), and no nystagmus. Axial length of the eyes was strongly elongated: (28.88–28.93 mm). Intraocular pressure was within normal limits (13 and 17 mmHg: IcarePRO (Icare Finland Oy); measurement under general anesthesia). Intraocular examination of both eyes showed extensive subepithelial corneal deposits, microcoria poorly responsive to mydriatics and a clear central lens.

**Fig. 2:**
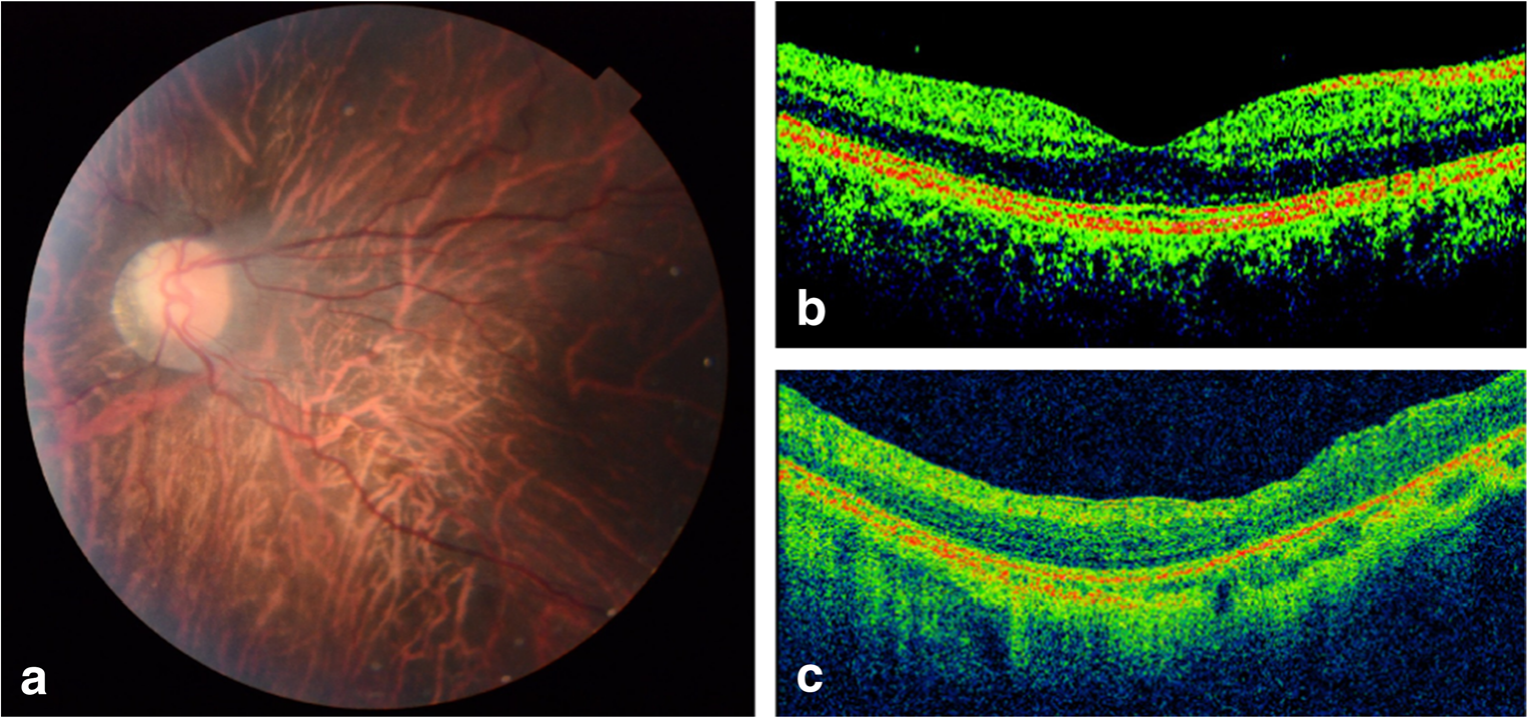
Ophthalmological findings in case 2: fundusphotography and cross-sectional (B-scan) detailed imaging of the macula, performed with spectral domain (SD)-OCT (Topcon 3D OCT-2000 device) **a** Left eye in case 2: the fundus shows diffuse tessellation of the retina, a mild waxy appearance of the optic nerve surrounded by a large scleral crescent. The macular reflex is absent, retinal vessels are prominently stretched. **b** Optic coherence tomography (OCT) of a healthy child: normal appearance of fovea, retinal contour, and thickness. OCT uses reflected light to form a cross-sectional image of the retina by automatic analysis of the reflective properties of retinal tissue. **c** OCT of the left eye of case 2: in the central area of the macula, a normal foveal contour is absent and retinal thickness is reduced (214 versus 245 μm average for age of 6 years). The retina shows a diffuse irregular contour. The scan is of limited quality due to poor cooperation, therefore individual analysis of retinal layers could not be performed

The subepithelial corneal deposits (bandkeratopathy) were presumed to be caused by calcium particle accumulation due to long-term undertreated renal failure with hypercalcemia and hyperphosphatemia. Surgical removal of the keratopathy showed improvement of vision to Logmar 0.8 Snellen 20/80, in both eyes. The cornea remained clear during follow-up. Fundoscopy showed a tessellated fundus, absence of the foveal reflex and a yellow, mildly waxy appearance of the optic disk. Retinal vessels were prominently stretched (Fig. 2a). Optic Coherence Tomography (OCT; Fig. 2c), showed a diffusely reduced thickness of the posterior pole and absence of a normal foveal contour. The central thickness of the foveal area was 214 μm (Fig. 2c). The scan was of suboptimal quality due to poor cooperation. Electroretinography was not performed.

Like her sister, she was assisted with low-vision devices and special training for low vision. Also this girl showed appropriate weight gain following initiation of gastrostomy feeding. In the months after admission, she underwent extensive neurological evaluation because of chronic severe headache attacks. Consecutive neurological examinations revealed no abnormalities. Because of the severity of symptoms, a brain MRI was performed 6 months after presentation, which showed aspecific subcortical and periventricular white matter lesions, without other structural abnormalities, thus non-explanatory for the symptoms. The headache attacks gradually improved over time.

Both sisters showed normal psychomotor development in the months after admission, with school results appropriate for age and acquirement of the Dutch language within 1 year. Neither parents showed evidence of ocular or renal problems. And, per anamnesis, no other family members were affected.

### DNA results

For the eldest sister, DNA mutational analysis for the *NPHS2* and *WT1* genes was performed in Syria in 2010 and revealed a single heterozygous nucleotide change in the *NPHS2* gene, which was classified a polymorphism and non-explanatory for the phenotype; exons 8 and 9 of the *WT1* gene did not reveal possible pathogenic mutations. *LAMB2* mutational analysis was not performed/reported.

**Fig. 3:**
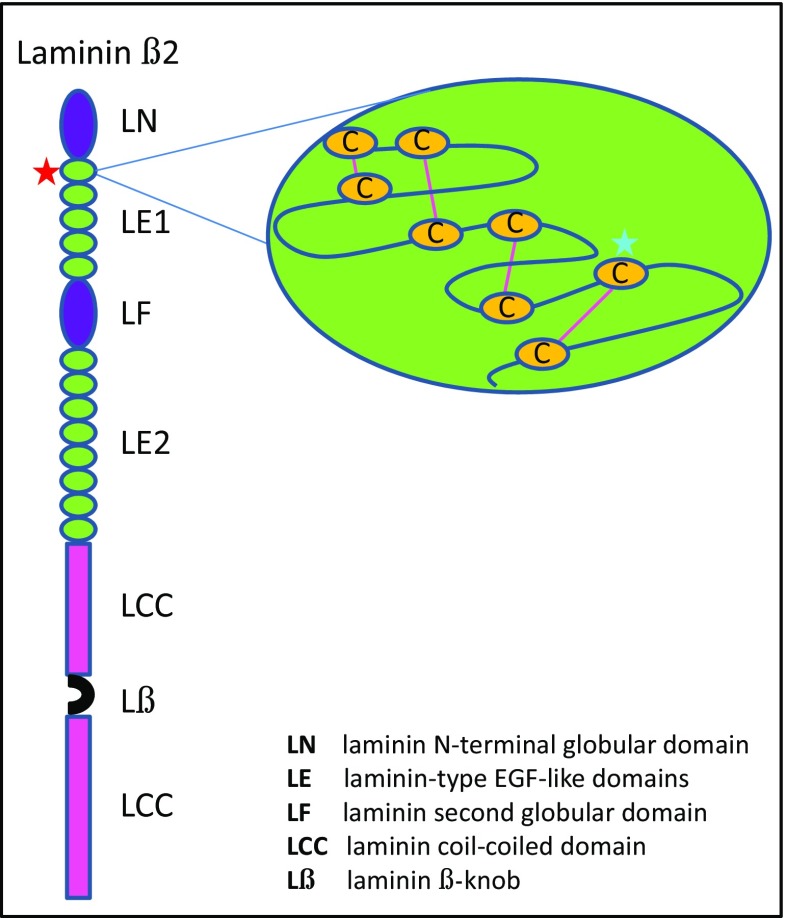
Schematic drawing of the functional domains of the human laminin β2 chain. The *red asterisk* indicates the position of the c.970T>C p.(Cys324Arg) mutation found in our patients. The first EGF-like module is depicted on the right; the *blue line* represents the amino acid sequence with only the highly conserved cysteine residues shown in *yellow*. The *pink connecting lines* indicate disulfide bonds. The light *blue asterisks* in the right panel indicates the position of the mutated seventh cysteine residue, which is replaced by arginine; p.(Cys324Arg)

Diagnostic DNA sequence analysis by means of massive parallel sequencing performed at the department of Genetics of the UMC Utrecht, revealed that both girls showed a homozygous c.970T>C p.(Cys324Arg) mutation in the *LAMB2* gene. This missense mutation located in exon 8 of the *LAMB2* gene, is situated within the first EGF-like module of the laminin β2 protein, and affects one of the highly conserved cysteine residues (Fig. 3). No other pathogenic mutations were observed in the nephrotic syndrome associated gene panel (genes: *CD151*, *COQ2*, *GLA*, *INF2*, *ITGA3*, *LMX1B*, *NPHS1*, *NPHS2*, *PDSS1*, *PDSS2*. *PLCE1*, *PTPRO*, *SCARB2*, *SMARCAL1*, *TRPC6*, *WT1*).

## Discussion

This report describes a novel mutation in exon 8 of *LAMB2*, the gene associated with Pierson syndrome and its milder variants [1, 2, 4]. The reported c.970T>C p.(Cys324Arg) variant has previously not been described in patients and was absent in >60.000 controls (http://exac.broadinstitute.org). The cysteine residue on position 324 is strongly conserved and lies within the first EGF-like module of the laminin β2 protein. This module contains eight conserved cysteine residues, which form disulfide bonds in order to achieve a correct 3D structure. The cysteine residue on position 324 is involved in the fourth disulfide bond in the EGF-like module (Fig. 3). Mutation of a nearby cysteine in the *LAMB2* gene (position 321; compound heterozygous with a *Leu*1393*Phe* and *Asn*1380*Lys* mutation) is a known pathogenic mutation and has been shown to be involved in the third disulfide bond of the domain [3, 4]. Reported patients presented with congenital nephrotic syndrome and ocular abnormalities consisting of nystagmus, myopia, strabismus, and hypopigmented retina. Additionally, a p.(Cys310Arg) mutation was found by mutational screening of a study population of steroid-resistant nephrotic syndrome [10], underscoring the importance of cysteines in this protein domain. Mutations in the *LAMB2* gene described thus far comprise missense, nonsense and splice site mutations, as well as small deletions and insertions, found either as homozygous or compound heterozygous sequence changes [4]. Genotype-phenotype correlation studies have revealed a significantly earlier manifestation (nephrosis <3 months of age) and worse prognosis (ESRD <1 year of age) of the renal phenotype in truncating *LAMB2* mutations (functional null alleles), compared to missense mutations (possibly hypomorphic alleles) [2, 4]. Regarding the ocular manifestations, almost all patients harboring bi-allelic nonsense or frameshift mutations exhibited microcoria in association with variable ocular abnormalities [9], while patients without microcoria showed variable mutation types [4]. With regard to the neurological phenotype, a previous genotype-phenotype study found 22 of 42 patients with *LAMB2* mutations who underwent neurological evaluation, to have neurodevelopmental deficits consisting of significant hypotonia/muscle weakness/myasthenia, significant motor delay and suspected or proven cognitive defects. These neurodevelopmental deficits were not clearly associated with a specific mutation type [4]. These observations show that although there is evidence for a genotype-phenotype correlation with respect to the renal and ocular phenotype in *LAMB2* mutations, the neurodevelopmental manifestations remain clinically variable and thus unpredictable.

The herein described c.970T>C p.(Cys324Arg) variant is considered a pathogenic missense mutation which probably results in disturbed formation of the fourth disulfide bond and hence disturbed 3D structural conformation of the laminin β2 protein. The aberrant protein presumably contains residual function (hypomorphic allele) and hence leads to a milder phenotype compared to the classic Pierson Syndrome [2].

The currently reported mutation corresponds to a phenotype with therapy-resistant nephrotic syndrome at the age of 18 months, leading to progressive renal failure, with end-stage renal failure within 4–5 years, accompanied by ocular abnormalities consisting of high myopia, microcoria, thinning of the retina with absence of the foveal reflex (*n* = 1) and optic nerve abnormalities (*n* = 1).

Importantly, the newly reported *LAMB2* c.970T>C p.(Cys324Arg) mutation was associated with normal neurological development in both siblings. The white matter lesions found on brain MRI in the youngest girl might be due to vascular changes as a consequence of her longstanding therapy-resistant hypertension. Both girls currently attend primary school and show age-appropriate school results. They were able to fully acquire the Dutch language within 1 year.

This report presents the variability of the renal, ocular, and neurological phenotypes associated with *LAMB2* mutations [4, 11], and underscores the importance of ophthalmologic examination in all children with unexplained renal insufficiency or nephrotic syndrome.

### Authors’ contributions

All authors contributed to the current manuscript according to their medical specialty (pediatric nephrology, ophthalmology, genetics and pathology):

F. Falix and M. Oosterveld: pediatric nephrology; introduction, clinical descriptions, discussion and conclusion

C. Bennebroek and R. Lapid-Gortzak: ophtalmology; ophtalmological findings, description and imaging, discussion and conclusion

B. van der Zwaag: human genetics; genetic analysis, and discussion

S. Florquin: pathology: pathological findings, interpretation, and description
